# Impairing the function of MLCK, myosin Va or myosin Vb disrupts Rhinovirus B14 replication

**DOI:** 10.1038/s41598-017-17501-z

**Published:** 2017-12-07

**Authors:** Antonio Real-Hohn, D. William Provance, Rafael Braga Gonçalves, Caio Bidueira Denani, Andréa Cheble de Oliveira, Verônica P. Salerno, Andre Marco Oliveira Gomes

**Affiliations:** 1 Instituto de Bioquímica Médica Leopoldo de Meis, Universidade Federal do Rio de Janeiro, Rio de Janeiro, Brazil; 2 Departamento de Biociências da Atividade Física, Escola de Educação Física e Desportos, Universidade Federal Rio do Janeiro, Rio de Janeiro, Brazil; 3 Center for Technological Development in Health, National Institute of Science and Technology for Innovation in Diseases of Neglected Populations, Oswaldo Cruz Foundation/Fiocruz, Rio de Janeiro, Brazil; 4 Departamento de Bioquímica, Instituto Biomédico, Universidade Federal do Estado do Rio de Janeiro, Rio de Janeiro, Brazil; 5 Instituto Nacional de Ciência e Tecnologia de Biologia Estrutural e Bioimagem, Rio de Janeiro, RJ Brazil

## Abstract

Together, the three human rhinovirus (RV) species are the most frequent cause of the common cold. Because of their high similarity with other viral species of the genus *Enterovirus*, within the large family *Picornaviridae*, studies on RV infectious activities often offer a less pathogenic model for more aggressive enteroviruses, e.g. poliovirus or EV71. Picornaviruses enter via receptor mediated endocytosis and replicate in the cytosol. Most of them depend on functional F-actin, Rab proteins, and probably motor proteins. To assess the latter, we evaluated the role of myosin light chain kinase (MLCK) and two myosin V isoforms (Va and Vb) in RV-B14 infection. We report that ML-9, a very specific MLCK inhibitor, dramatically reduced RV-B14 entry. We also demonstrate that RV-B14 infection in cells expressing dominant-negative forms of myosin Va and Vb was impaired after virus entry. Using immunofluorescent localization and immunoprecipitation, we show that myosin Va co-localized with RV-B14 exclusively after viral entry (15 min post infection) and that myosin Vb was present in the clusters of newly synthesized RNA in infected cells. These clusters, observed at 180 min post infection, are reminiscent of replication sites. Taken together, these results identify myosin light chain kinase, myosin Va and myosin Vb as new players in RV-B14 infection that participate directly or indirectly in different stages of the viral cycle.

## Introduction

The common cold is normally limited to the upper respiratory tract^1^. The economic costs associated with non-influenza respiratory infections have been estimated to approach 40 billion dollars per year in the United States alone^2^. While various viruses can cause symptoms of a common cold, about 50% of all the cases are caused by human rhinoviruses (RVs), members of the family *Picornaviridae* in the genus *Enterovirus* ^3^. When associated with other pathogens or conditions such as asthma, chronic obstructive pulmonary disease or cystic fibrosis, RV infections can lead to severe and life threatening airway diseases^4^. Due to the high similarity presented by the thirteen species of the genus *Enterovirus* (including rhinovirus genera A, B, and C) with respect to their genome sequences, the 3D-structure of their protein shells and based on observations with poliovirus, it is believed that the release of the RNA genome from a virion only occurs after internalization or at least after the virus becomes sheltered from the extracellular medium^5^.

Following entry by receptor-mediated endocytosis along diverse pathways^6–9^, the virus first converts into the subviral A-particle that is expanded by 4% with respect to the native virion. The conformation adopted is more permeable for the exit of the RNA genome, probably through one of the pores on the 2-fold axes or at the pseudo 3-fold axes^10–13^. Cryo-electron microscopy image reconstructions of heated poliovirus suggest that the highly structured RNA unfolds and starts exiting as a single-strand though one of the holes along the 2-fold axes^14^ leaving an empty capsid (subviral B-particle), which might then be shuttled to lysosomes for degradation^15^. Snug attachment of the viral capsid to the endosomal membrane might establish a tight seal^16^ allowing the genome to cross the lipid bilayer without exposure to the degradative milieu of the (late) endosome^17^.

The viral RNA appears in the cytosol as soon as 10 min after the initiation of infection^18^. Recently, it was demonstrated that the genomic RNA of poliovirus remains RNAse-protected during exit from the capsid^19^ suggesting a specific mechanism of viral genome delivery that not only directs, but also protects the RNA such that it reaches the cytoplasm intact and functional. Despite intense studies with several drugs targeting host proteins/mechanisms, this early step in infection is not fully understood. Compounds that increase endosomal pH have proven very effective at interfering with a majority of RVs^20–22^, different intracellular routes are likely to be used^23^. Internalization of RV-A2 and RV-B14 were reduced in cells expressing dominant-negative dynamin II (K44A)^24–27^ probably due to disturbance of proper clathrin-coated pit maturation^28^. Regardless of actin dependence of RVs^9,29^, RV-A2, RV-A16, RV-B3, and RV-B14 infections were not impaired in nocodazole treated cells^8,18,21,30^. Nonetheless, RV-B89 infection seems to be different, as ciliobrevin and nocodazole can reduce infection^8,9^.

The current paper reports that RV-B14 infection requires myosin light chain kinase (MLCK) for cell entry while myosin Va and Vb are required for intracellular events prior to replication. MLCK possesses a critical role in regulating the cell membrane tension and protrusion, thereby stabilizing the membrane skeleton through F-actin-binding^31^. The molecular motors myosin Va and Vb are described as complementary intracellular cargo transporters^32^. Myosin Va can transport endogenous mRNA^33^ and various intracellular vesicles^34^. Myosin Vb, on the other hand, is involved with endocytic membranes recycling^35^. Therefore, we propose that RV-B14 uses a “phagocytic-like” mechanism for entry due to its MLCK dependency, intracellular degradation evasion in dominant-negative expressing cells (Va and Vb) and the presence of RV-B14 (protein and RNA) in structures similar to a phagocytic cup in the initial steps of infection. Further, myosin Va appears to have an early role in replication and myosin Vb a late role.

## Results

### 1-(5-Chloronaphthalenesulfonyl)homopiperazine-HCl (ML-9) impacts RV-B14 entry

**Figure 1:**
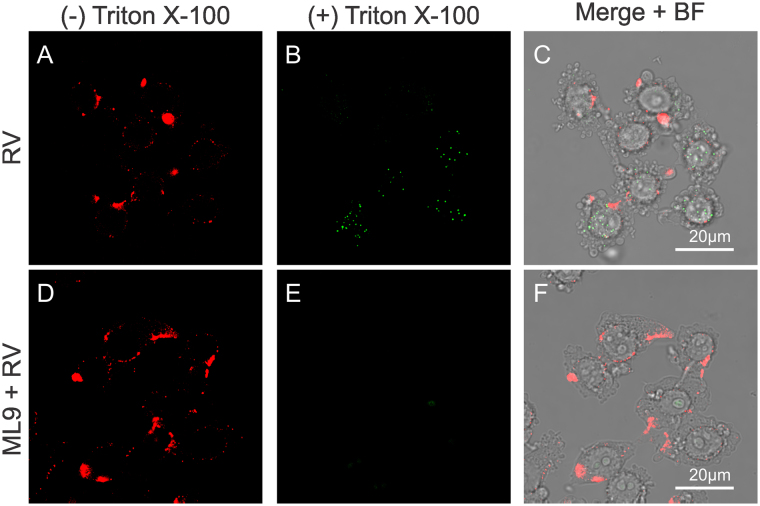
ML9 impacts on RV-B14 entry. Cells were incubated without (**A–C**; control) or with 10 µM ML9 (**D**–**F**) for 30 min prior to infection with RV-B14 (MOI = 10) for 1 h at 34 °C. Cells were processed for confocal microscopy before and after permeabilization with Triton X-100 as indicated. Extracellular (**A** and **D**) and intracellular (**B** and **E**) viruses were visualized with anti-RV-B14 plus IRDye® 680LT anti-Rabbit IgG (red) and anti-RV-B14 plus anti-Rabbit IgG H&L (FITC, green), respectively. Juxtaposition of images after permeabilization with Triton X-100 (**B** vs **E**) reveals that ML9 treatment prevents RV-B14 entry.

Wortmannin blocks phosphatidylinositol 3-kinases (PI3K) and delays infection of RV-A2 after entry by retarding the transfer of virions from early to late endosomes^36^. However, wortmannin can also impair MLCK function by directly binding at or near to the catalytic site of the enzyme in an irreversible manner^37^. Furthermore, MLCK is a calmodulin activated kinase responsible for myosin light chain phosphorylation, thus modulating myosin II binding to F-actin, and is described to be essential in endocytic routes in non-muscular cells^38^. To study whether MLCK might also be involved in RV-B14 entry, cells were pretreated with 10 µM ML9 30 min before infection. ML9 is a very specific MLCK inhibitor, structurally different from wortmannin, that binds at or near the ATP-binding site causing a competitive inhibition of the kinase^39^. Sixty minutes post infection (PI), cells were fixed and labeled with anti-RV-B14 followed by IRDye® 680LT conjugated anti-Rabbit IgG (Red). Next, cells were permeabilized and again labeled with anti-RV-B14, but followed by fluorescein isothiocyanate (FITC) conjugated anti-Rabbit (Green) to distinguish extracellular (plasma membrane-bound) and intracellular virus^40^. ML-9 pretreatment significantly reduced RV-B14 entry (Fig. 1). This effect differed from that observed for wortmannin in RV-A2 infection and could be explained by different sites of action of wortmannin^36^ and ML-9.

### Expression of myosin Va or Vb tail domains prevents RV-B14 propagation

**Figure 2:**
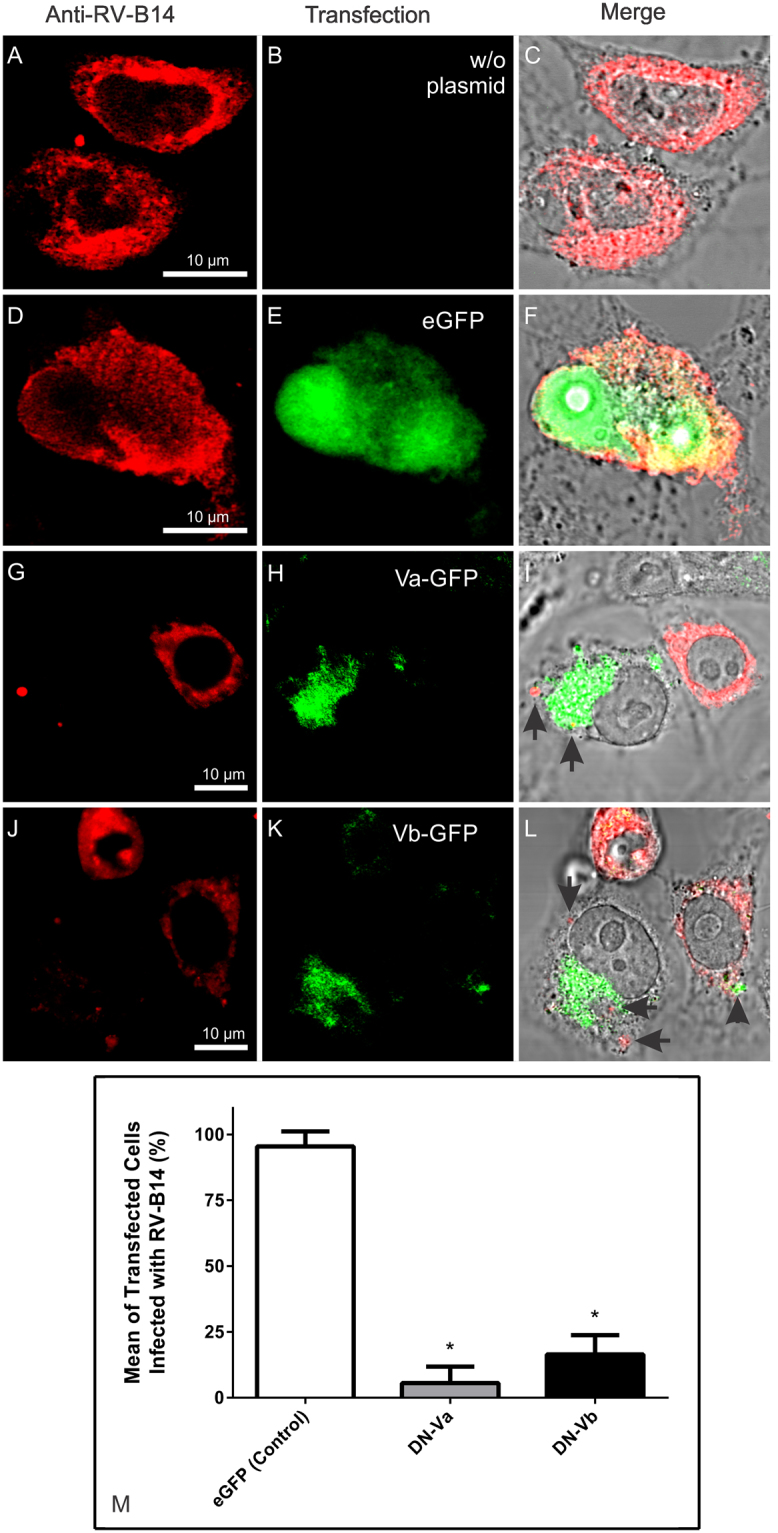
Overexpression of myosin Va or Vb tail domains prevents virus infection. HeLa-H1 cells transfected with plasmids encoding dominant-negative forms of myosin Va (DN-Va) or myosin Vb (DN-Vb), fused to eGFP, were infected with RV-B14 (MOI = 1) for 20 h at 34 °C. Mock transfected cells (w/o plasmid) and cells transfected with an eGFP plasmid alone (eGFP) were similarly infected. In the upper panel a representative field for each transfection is shown (eGFP expression in green): mock (**A**–**C**); eGFP (**D**–**F**); DN-Va (**G**–**I**), and DN-Vb (**J**–**L**). After infection, cells were fixed and labeled with anti-RV-B14 plus anti-Rabbit IgG H&L (TRITC, red). Fluorescence images were merged with bright field images for better distinction of cells boundaries (**C,F,I** and **L**). RV-B14 particles persisted at the periphery of cells expressing DN-Va or -Vb, (arrows in **I** and **L**, respectively). Note that some cells present low expression of DN-Vb (arrowhead in **L**). (**M**) Quantification of transfected cells infected with RV-B14 (% of transfected cells in 10 different fields). *p value < 0.0001 (vs. control).

Initial evidence for the importance of myosin V in viral infections came from the mild inhibition of respiratory syncytial virus in MDCK cells constitutively expressing the myosin Vb tail from a doxycycline controlled cassette^41^. Indeed, myosin V is involved in intracellular cargo trafficking^42,43^. To evaluate the potential roles of myosin Va and Vb function in RV-B14 infections, dominant-negative mutants of each fused to eGFP (DN-Va and DN-Vb) were obtained. These GFP-tail fusion proteins possess the cargo-binding motifs without the motor domain, and can compete for cargo binding with the respective endogenous full-length myosin V, disrupting its transport^44^. In addition, the DN-Va construct contained the alternatively spliced DDK exon, which can be bound by dynein light chain, and allow DN-Va to sequester this adaptor protein^45^. HeLa-H1 cells were transfected with individual constructs (DN-Va or DN-Vb) and infected with RV-B14 (wild type without labeling) at 34 °C for 20 h. Control experiments demonstrated that neither mock transfection (Fig. 2A–C) nor transfection with a plasmid encoding eGFP alone (Fig. 2D,E) interfered with RV-B14 infection (Fig. 2, Anti-RV-B14 column). In contrast, cells expressing either DN-Va (Fig. 2G–I) or DN-Vb (Fig. 2J–L), which are fused to eGFP, were virtually unaffected by RV-B14 infection. Nevertheless, endocytosed virus particles were observed in the cells transfected with DN-Va or DN-Vb (Fig. 2I and L; arrows). Further, we noted that infections by RV-B14 could occur in cells with the lowest observable expression of DN-Vb (Fig. 2L; arrow head). The number of cells doubly positive for eGFP and viral protein were analyzed in 10 different fields (Fig. 2M), which revealed that the presence of eGFP had no effect on viral replication; nearly 100% of the cells expressing eGFP were also positive for RV-B14 proteins. In contrast, expression of either DN-Va or DN-Vb (Fig. 2M) had a significant impact on virus infection (*p value < 0.0001).

### Myosin Va colocalizes with viral protein and BrU-RNA in phagocytic cup-like structures

**Figure 3:**
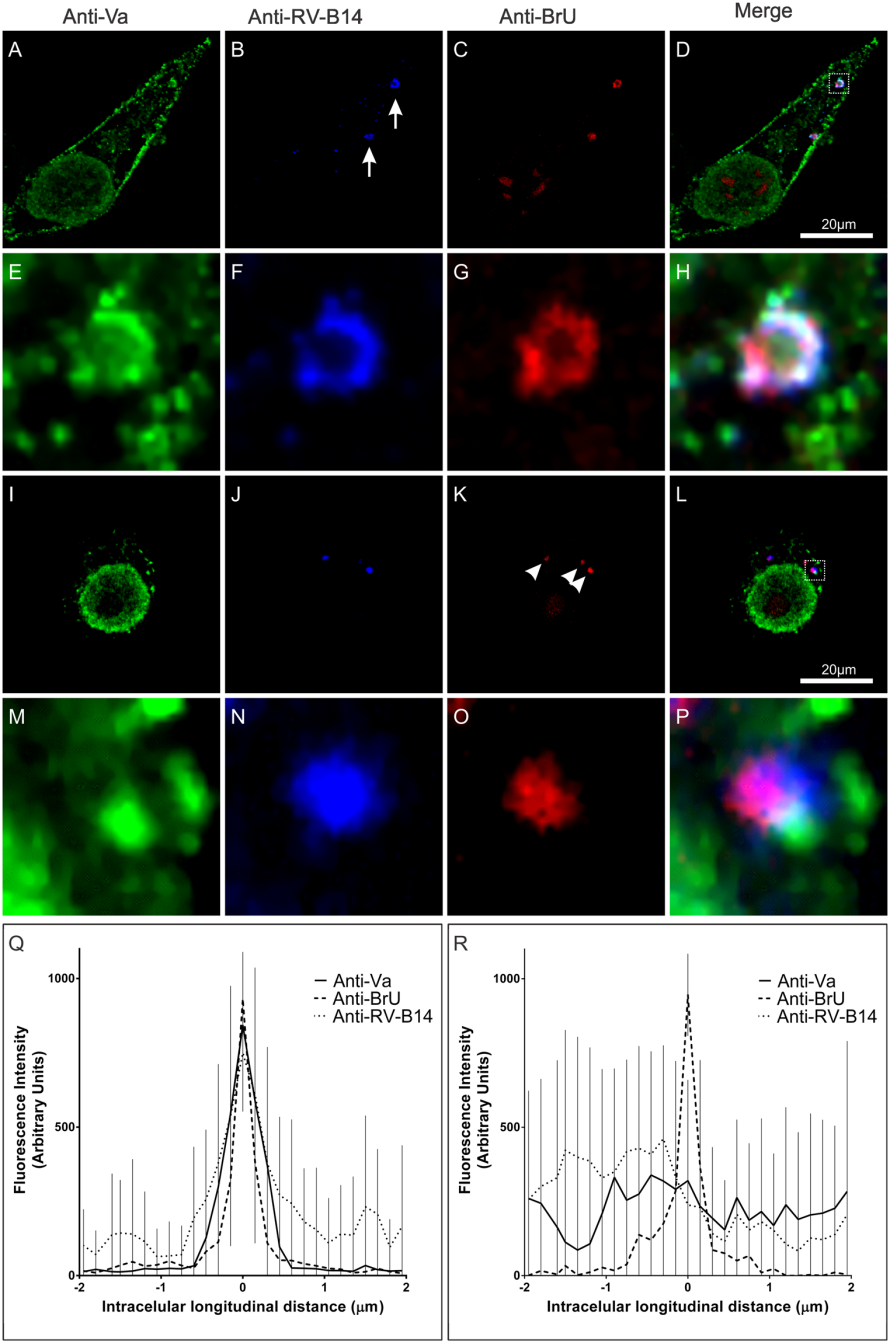
Myosin Va colocalizes with viral BrU-RNA containing vesicle in an early phase of infection. HeLa-H1 cells infected with BrU-RV-B14 (MOI = 10) for 15 min (**A–H**) or 60 min (**I**–**P**) at 34 °C. After the infection, cells were labeled for anti-myosin Va (**A,E,I**, and **M**; anti-Va - green), anti-RV-B14 (**B,F,J**, and **N**; anti-RV-B14 - blue), and anti-BrU-RNA (**C,G,K**, and **O**; anti-BrU - red). (**D,H,L**, and **P**) Merged images demonstrating the intracellular disposition of myosin Va, RV-B14 protein, and BrU-RNA. (**E–H** and **M–P**) Enlarged regions correspond to the dashed boxes in panels **D** and **L**, respectively. (**B**; arrows) Internalized viral particles present in the periphery of the cell. (**K**; arrowheads) Perinuclear BrU-RNA foci. (**Q** and **R**) Intensity histogram of signals (10 different experiments) collected along a line (4 µm long and 0.1 µm wide centered on the anti-BrU maximum intensity) for 15 and 60 min, respectively.

To study the temporal aspects of myosin Va function potentially contributing to RV-B14 infections, the position of viral RNA and viral protein in relation to endogenous myosin Va was determined at 15 min and 60 min PI. To specifically detect virus genomes, viral RNA was metabolically labeled with bromouridine (BrU)^5,46,47^ during virus stock production. The BrU labeling showed no impact on the infectivity of purified virus. HeLa-H1 cells were infected with the BrU-labeled RB-14 (BrU-RV-B14) for either 15 min (Fig. 3A–H) or 60 min (Fig. 3I–P) before fixation to separately visualize viral protein, viral RNA, and myosin Va with suitable antibody combinations. At 15 min PI, RV-B14 protein accumulated in small dots and clusters (Fig. 3B; arrows), most of which coinciding with viral RNA (Fig. 3C). Colocalization of the myosin Va signal (Fig. 3A and D) with both RV-B14 protein and BrU-RNA was immediately apparent, and could be even better distinguished at higher magnification (Fig. 3E–H; inset of D dashed square). The subcellular distribution of myosin Va coincided spatially with both the RV-B14 RNA genome and protein (Fig. 3D and H). The morphological shape of the labeled structures was reminiscent of phagocytic cups, a protrusion of the cell surface that extends along and finally closes around the particle or nutrient to be engulfed^48^. At 60 min PI, multiple viral RNA foci (Fig. 3K; arrowheads) were detected in the perinuclear region (Fig. 3L). However, the colocalization of myosin Va, RV-B14 protein and BrU-RNA signals was less clear, even at higher magnification (Figs. 3M–P; inset of L, dashed square). A signal intensity histogram was measured for the anti-myosin Va (anti-Va), anti-RV-B14, and the anti-BrdU antibody (anti-BrU) localizations along a 4 µm line that was 0.1 µm wide and centered on the region with the maximum anti-BrU intensity (examples in Supplementary Fig. S1). Fluorescent signals from 10 different experiments were grouped and plotted for the experiments performed at 15 (Fig. 3Q) and 60 min PI (Fig. 3R). The intensities of the peaks were well matched at 15 min PI, but not at 60 min. From this analysis, we conclude that, at 15 min PI, vesicles contain virus capsids and partially released RNA bound to myosin Va, and that RNA is fully accessible to translation and replication at 60 min PI.

### BrU-labeled viral genomes co-precipitate with myosin Va in a time-dependent manner

**Figure 4:**
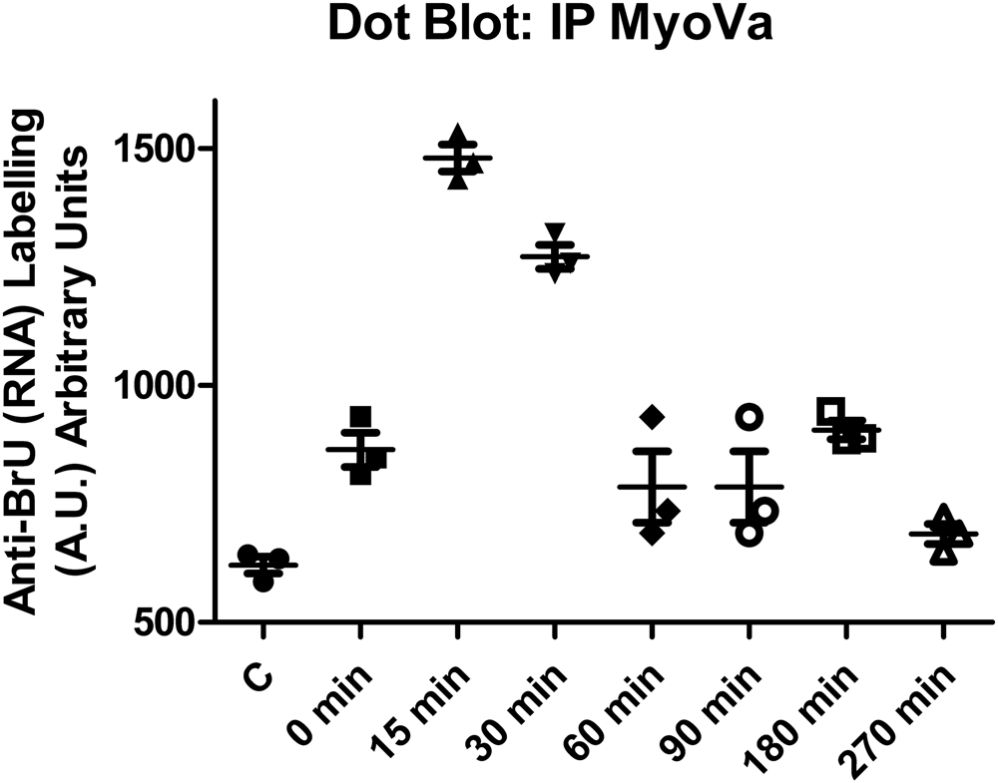
BrU labeled viral genomes co-precipitate with myosin Va in a time-dependent manner. Hela-H1 cells were infected with BrU-RV-B14 (MOI = 10) at 0, 15, 30, 60, 90, 180, and 240 minutes. Cells were lightly crosslinked (1% formaldehyde for 2 min) and lysed with RIPA buffer. Extracts were centrifuged and 4 h incubated with (mouse) anti-myosin Va antibody followed by overnight incubation with anti-Mouse antibody linked to agarose beads. Clarified complexes were immobilized on PVDF membrane, probed with (rabbit) anti-BrdU antibody (plus IRDye® 800LT anti-Rabbit IgG), striped, and probed with (mouse) anti-myosin Va antibody (plus IRDye® 680LT anti-Mouse IgG). Quantification from three independent experiments (different symbols in the graph) showing the signal intensity of anti-BrU (normalized by anti-myosin Va signal) at different times PI.

To assess whether myosin Va directly or indirectly interacts with the RV-B14 genome, HeLa-H1 cells were challenged with BrU-RV-B14 (similar to Fig. 3), and the infection was halted at different times by crosslinking with formaldehyde^49^. Cells were subsequently lysed with RIPA buffer and myosin Va was immunoprecipitated with antibodies bound to agarose beads. The presence of BrU-RNA was then ascertained on a dot-blot with anti-BrdU antibody and the quantity of BrU-RNA, relative to the anti-Va signal, was plotted against time (Fig. 4). The BrU-RNA signal was highest at 15 min, decreased at 30 min, and returned to near control levels at later times. These findings corroborate the colocalization data (Fig. 3), as RV-B14 RNA release appears to be highly coincident with the presence of myosin Va. This suggests that myosin Va could contribute to the transport and proper localization of the viral RNA genome from the cell periphery to the replication sites, similar to its previously described role in mRNA transport and proper localization in dendritic spines^33,50^.

### Myosin Va is absent from newly synthesized RNA clusters in late events during RV-B14 infection

**Figure 5:**
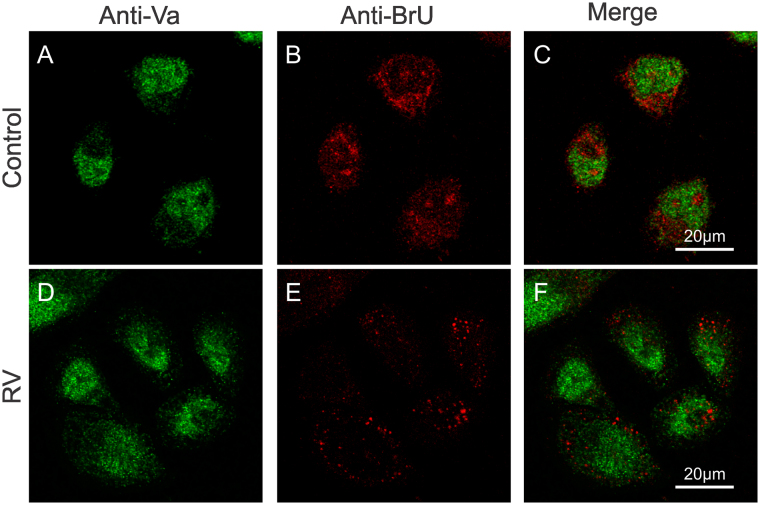
Identification of putative RV-B14 replication sites. HeLa-H1 cells were mock infected (**A**–**C**) or infected (**D**–**E**) with RV-B14 (MOI = 50) for 180 min at 34 °C. At 30 min PI, cells were pulse labeled with 2.5 mM BrU to incorporate BrU into nascent RNA. The cells were then processed for confocal microscopy with (rabbit) anti-myosin Va antibody plus anti-Rabbit IgG H&L (FITC) (**A** and **D**; green) and Alexa Fluor 555® anti-BrdU (**B** and **E**; red). (**B**) Nascent BrU-RNA. (**E**) BrU-RNA clusters presumably representing viral RNA. (**C** and **F**) Merged images from (**A,B** and **D–E**) respectively.

To study the onset of RV-B14 replication, we examined the distribution of newly synthesized RNA at 180 min PI. In previous reports, the *de novo* synthesis of RV RNA genomes was detected in HeLa cells through fluorescent *in situ* hybridization and ^3^H-uridine incorporation within 180–210 min PI^18,51^. Here, mock-infected controls (Fig. 5A–C) and RV-B14 (wild type without labeling) infected cells (Fig. 5D–F) were pulse-labeled with BrU at 30 min PI until 180 min PI. Subsequently, the cells were fixed, labeled with anti-myosin Va (Fig. 5A,C,D, and F; green) and anti-BrU (Fig. B,C,E, and F; red). At 180 min PI, the distribution of myosin Va was similar between the control (Fig. 5A) and infected cells (Fig. 5D) with no evidence of its colocalization with the BrU signal (Fig. 5C and F), restricting our proposed role for myosin Va to the initial steps of RV-B14 infection.

The observations of the intracellular BrU localization revealed that in mock infected cells, the BrU-labled RNAs were evenly distributed through the cytosol (Fig. 5B). On the other hand, in RV-B14 infected cells, the BrU-RNAs were concentrated in clusters unevenly distributed through the cytosol (Fig. 5E). The observed pattern of the *de novo* synthesized RNA in infected cells was compatible with previously reported structures for rhinovirus that were defined as replication sites (see Fig. 1 in Jurgeit, *et al*.^52^). Since specific markers for rhinovirus replication sites were not employed, we refer to this pattern of RNA clusters, which was both time and infection-state dependent, as putative RV-B14 replication sites.

### Myosin Vb is present in putative RV-B14 replication sites

**Figure 6:**
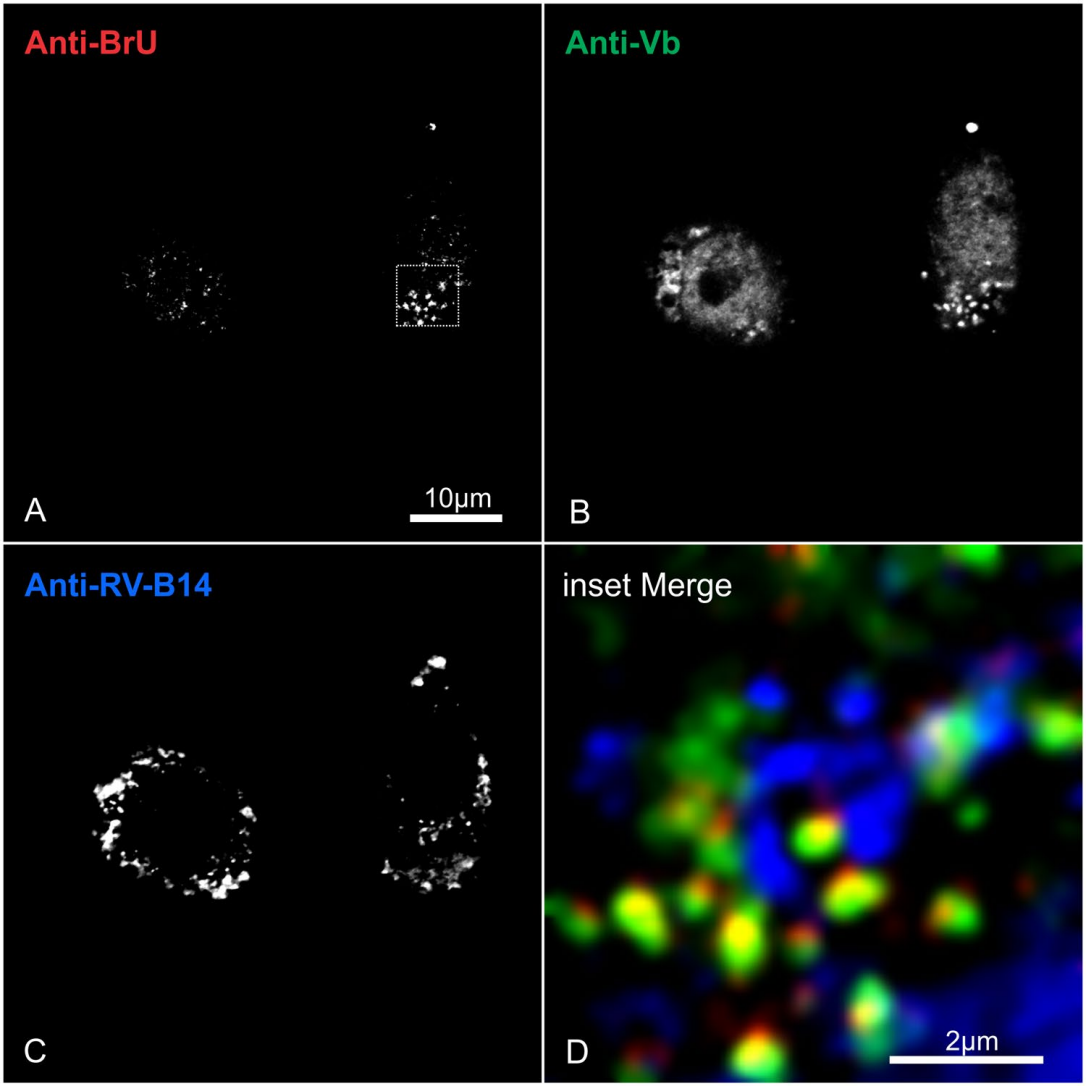
Putative RV-B14 replication sites recruit myosin Vb protein. HeLa-H1 cells were infected with RV-B14 (MOI = 50) for 180 min at 34 °C. At 30 min PI, cells were pulse labeled with 2.5 mM BrU to incorporate it into nascent RNA. Cells were subsequently processed for confocal microscopy with Alexa Fluor 555® anti-BrdU (**A**; red), (mouse) anti-myosin Va antibody plus Alexa Fluor 488® goat anti-Mouse (**B**; green) and (rabbit) anti-RV-B14 plus IRDye® 680LT anti-Rabbit IgG (**C**; blue). (**D**) Enlargement of dashed box in A.

Previous data on the localization of myosin Vb to nuclear transcription centers^53^ led us to hypothesize that myosin Vb could be sequestered during RV-B14 infection to compose the cytosolic replication complex. To test this hypothesis, HeLa-H1 cells were infected with RV-B14 (wild type without labeling) and pulse-labeled with BrU at 30 min PI (similar to Fig. 5). Cells were fixed at 180 min PI and examined for the distribution of the *de novo* synthesized BrU-containing RNA (Fig. 6A), myosin Vb (Fig. 6B), and of *de novo* synthesized RV-B14 capsid proteins (Fig. 6C). The signal distributions were better distinguished at a higher magnification (Fig. 6D inset of 6 A, dashed square). The RNA clusters induced by infection could be promptly identified (Fig. 6D – red, similar to Fig. 5E), and showed a strong colocalization with myosin Vb (Fig. 6D – green). Remarkably, we observed a well-defined crown of RV-B14 proteins surrounding the RNA clusters (Fig. 6D – blue), which supports our proposition that the structures labeled by BrU in infected cells represent RV-B14 replication sites (Fig. 5E).

**Figure 7:**
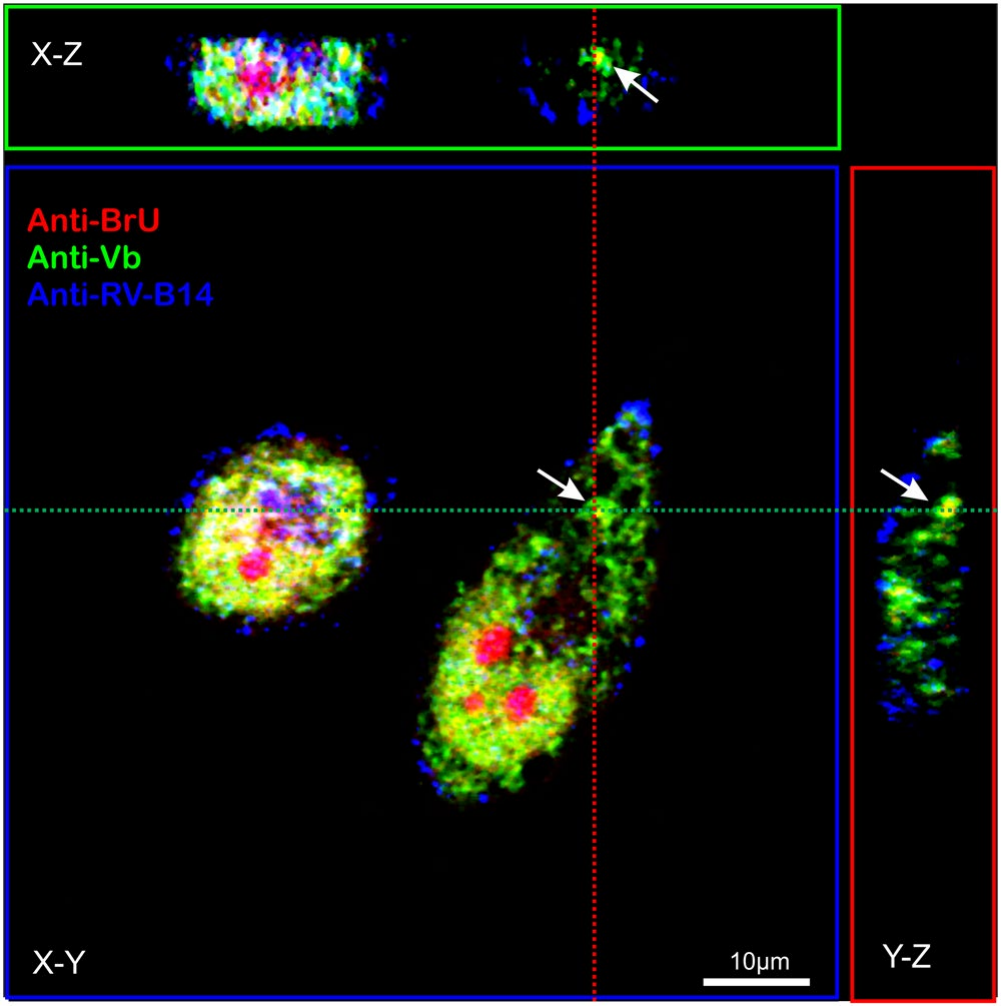
The three dimensional distribution of myosin Vb coincides with putative RV-B14 replication sites. HeLa-H1 cells were infected with RV-B14 (MOI = 50) for 180 min at 34 °C. At 30 min PI, cells were pulse labeled with 2.5 mM BrU to incorporate BrU into nascent RNA. At the end of infection, cells were then processed for 3D confocal microscopy with Alexa Fluor 555® anti-BrdU (anti-BrU; red), (mouse) anti-myosin Vb antibody plus Alexa Fluor 488® goat anti-Mouse (anti-Vb; green), and (rabbit) anti-RV-B14 plus IRDye® 680LT anti-Rabbit IgG (anti-RV-B14; blue). A representative cell was imaged with z-series (Zen Software – LSM510) to capture several image planes from the top to the bottom of the cell. The X-Z axis (green dashed line) was projected to the upper box (green) and Y-Z axis (red dashed line) was projected to the right-side box (red). Nucleolus was nonspecifically labeled with Alexa Fluor 555® anti-BrdU.

**Figure 8:**
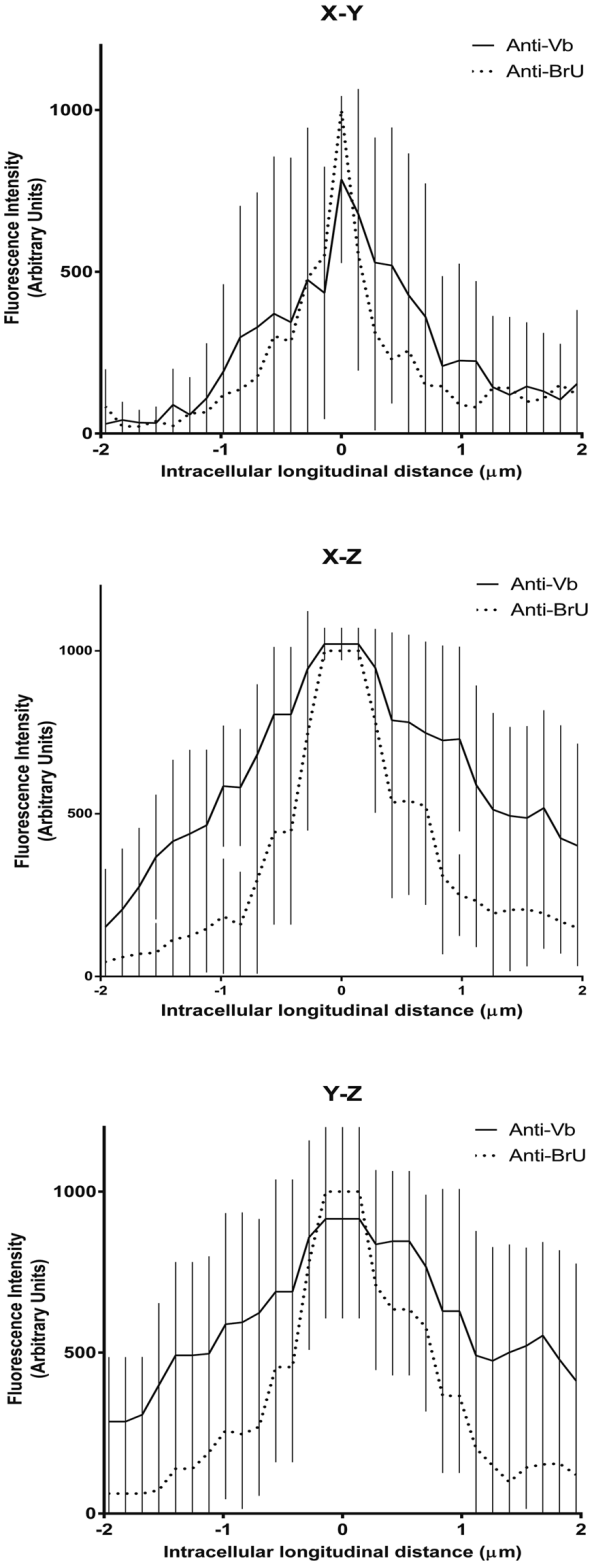
Fluorescence intensity 3D quantification confirms the presence of myosin Vb within putative RV-B14 replication sites. The intensity histogram of signals from BrU-RNA and myosin Vb (10 different experiments similar to Fig. 7) were measured along a line (4 µm long and 0.1 µm wide centered on the anti-BrU maximum intensity). Both individual fluorescent signal (anti-BrU and anti-Vb) were grouped and plotted for each axis. The plateau observed along the X-Z and Y-Z axis is due to lower microscope resolution in Z as compared to the resolution along the X-Y axis.

Three-dimensional imaging of a representative cell infected with RV-B14 (wild type without labeling) and pulse labeled with BrU 30 min PI (similar as Figs 5 and 6) revealed colocalization of myosin Vb with the putative RV-B14 replication sites in all three dimensions (Fig. 7, white arrow). To quantify the fluorescent signals of anti-BrU and anti-myosin Vb for comparison in 3D, we measured the fluorescence intensities along a line (4 µm long and 0.1 µm wide centered on the anti-BrU maximum intensity) from 10 different experiments, which were plotted in each dimension (Fig. 8). In all dimensions, the maximum intensity of myosin Vb correlated with the maximum BrU signal. This 3D colocalization suggested that myosin Vb was definitively present in the putative RV-B14 replication sites.

## Discussion

The MLCK inhibitor, ML9, affected RV-B14 entry (Fig. 1) to a greater degree than that observed previously with wortmannin during an RV-A2 infection^36^. Although not mentioned by Brabec *et al*., the RV-A2 inhibitory effect of wortmannin could also be explained by a MLCK inhibition^37^, in addition to the PI3K inhibition described in the report^36^. MLCK is, together with Rho-kinase, fundamental for stress fiber dynamics^54^, which can be exploited by viruses for their intracellular transport to the site of replication^55^. Furthermore, PI3K and MLCK inhibitors have been demonstrated to impair non-opsonic phagocytosis^56^ and phagocytosis in Kupffer cells^57^, respectively. Our observed inhibition of RV-B14 entry in the presence of ML9 strongly suggests a role for MLCK in virus internalization, most likely involving actin dynamics and phagocytosis-like mechanisms.

Rhinovirus infection strongly depends on cytoskeletal proteins^9^. Overexpression of the Rab11 mutants Rab11-GTP (constitutively active) and Rab11-GDP (a dominant-negative) revealed that uncoating of RV-A89 depends on Rab11^8^. Other Rab11 dependent viruses include type I parainfluenza virus^58^, respiratory syncytial virus^41^, and human immunodeficiency virus 1, which are also dependent on myosin Vb during infection^59^. Conzemius *et al*. demonstrated that RV-B14 infection progressed in cells expressing a dominant-negative Rab11^8^. This demonstrated that RV-B14 does not enter the endosome recycling pathway. However, RV-B14 infection could require other factors, including Rab5 and Rab7 to transit from early endosomes to late endosomes (reviewed by Hutagalung and Novick^60^), and a demand for myosin Vb was not excluded.

The myosin Vb requirement for type I parainfluenza virus and respiratory syncytial virus infection prompted us to study whether myosin Vs were also playing a role in RV-B14 infection. We began by determining whether myosin Va, which can interact differently with various Rab proteins^61,62^, has a role in RV-B14 entry. From the data shown in Fig. 2, it is clear that interfering with the function of myosin Va and/or Vb dramatically reduced RV-B14 replication, as deduced from the virtual absence of viral proteins in cells expressing the DN tail domains and challenged with RV-B14. Additionally, the internalized RV-B14 (Fig. 2I and L – arrows) appeared to remain intact, inferred by the shape of the fluorescent signal, and presumably infectious. Unfortunately, we were not able to test whether it was indeed infectious, because it was not possible for us to exclusively extract these particles. Interestingly, the intracellular preservation of RV-B14 in the presence of DN-Va resembled the *Mycobacterium tuberculosis* that persists within the host cells in the immature phagosomal compartment due to several evasion strategies^63^.

Next, we determined the distribution of RV-B14 structural proteins, its RNA genome, and endogenous myosin Va at different times PI by immunofluorescence microscopy. A major difficulty to overcome in these experiments was to specifically identify the viral genome with high sensitivity during the early stages of infection. Previously, fluorescence *in situ* hybridization probes were used to demonstrate that the RV genome became accessible in the cytoplasm 10–12 min after the virus was presented to the host cell^18^. Molecular beacons have also been used to track the RNA genome of another enterovirus, coxsackievirus B6, inside host cells^64^. However, both approaches require unique reagents and highly involved protocols. For molecular beacons, the background fluorescence from non-functional beacons can negatively impact the sensitivity of detection. Other probes have been attempted, but were generally ineffective for localizing the viral RNA after it reaches the cytosol^16^, most likely due to a dilution of the probe, intracellular competition with endogenous RNA, or degradation^18^.

Here, we used BrU to directly label the viral RNA genome metabolically during production of viral stocks. It did not interfere with replication, since viral stocks were readily generated (BrU-RV-B14). This approach allowed the incorporation of multiple BrU labels into each genome that appeared to provide high sensitivity in the identification of the RNA genome, specifically from the internalized virus after release from the capsid and arrival in the cytoplasm. This advancement was important to successfully observe the colocalization of myosin Va with the RV-B14 genome and the capsid proteins that occurred within 15 min PI (Fig. 3A–F). Similar kinetics were described in the recruitment of ceramide and glycosphingolipid to the cell membrane during RV-B14 infection, which showed an accumulation that peaked at 15 min PI^65^. The same ceramide and glycosphingolipids were also accumulated during *Pseudomonas aeruginosa*, *Staphylococcus aureus*, and *Neisseriae gonorrhoeae* infection^66–69^. Additionally, the shape of this BrU-RNA and RV-B14-protein containing structures resemble myosin Va phagocytic cups^70^.

By 60 min, myosin Va was no longer localized to the same subcellular regions as the RV-B14 BrU labeled genome and its capsid proteins; neither did the capsid proteins colocalize with the viral genome, indicating that (complete) uncoating had occurred (Fig. 3G–L), and capsid proteins could enter the degradation route (as seen for RV-A2 in Fig. 1 from Neubauer, *et al*.^22^). Quantification of the intensity histograms (Fig. 3M and N) corroborated the convergence of all three fluorescent signals at 15 min and their separation at 60 min PI. To further explore the interaction of all these components, we immunoprecipitated myosin Va from cells infected with BrU-RV-B14 (similar as in Fig. 3) and probed the material with antiserum against BrU (Fig. 4). The BrU-RNA peak at 15 min and the return to baseline at 60 min was consistent with the colocalization results (Fig. 3). The immunoprecipitation assay included a crosslink to stabilize protein-RNA complexes, and to prevent myosin Va from dissociating from the BrU-RV-B14 genome during the procedure^71^. Our observations of uncoated BrU-RNA (Figs 3 and 4) did not exclude the possibility that we actually detected A-particles with partially released RNA^72^. Nevertheless, our data strongly argue that myosin Va was playing an essential role in the release of the RV-B14 genome. The possible interaction between virus RNA and myosin Va, according to the immunoprecipitation assay (Fig. 4), could be direct or indirect. The indirect interaction could use dynein light chain 1 (DLC1) as an adaptor, similar to what was observed in drosophila, where DLC1 binding to *gurken* mRNA is essential for its proper localization in oocytes^73,74^. Another possibility is that the myosin plays a role in mechanical destabilization of the capsid^75,76^. None of these possibilities can be refuted without further investigation.

BrU incorporation into *de novo* synthesized RNA between 30 and 180 min PI allowed the identification of RNA clusters in infected cells (Figs 5–7). Approximately 1 hour PI, RVs express 2A^pro^, a protease that degrades eIF4G and polyA binding protein, to dismantle cellular cap-dependent translation that predominates for host mRNA. Viral translation continues through internal ribosome entry sites, which is a viral RNA-specific sequence that allows the recruitment of ribosomes in a cap-independent manner^77^. Furthermore, 2A^pro^ cleaves Phe/Gly-containing nucleoporin proteins within nuclear pore complexes, which impairs nuclear export^78^. Consequently, these groups of events could help to explain the differences observed in the patterns of *de novo* synthesized RNA (Fig. 5) between mock and infected cells, which we propose to represent RV replication sites in infected cells.

Different groups showed that replicated RV RNA genomes could be detected in HeLa cells through fluorescent *in situ* hybridization and ^3^H-uridine incorporation within 180–210 min PI^18,51^. We observed in our work that at 180 min PI, infected HeLa cells were presenting well-defined RNA clusters. These RNA clusters possess a size and intracellular distribution similar to those observed for replication sites from diverse enteroviruses^79,80^. Since our images do not include labeling for the viral replication machinery, we can only describe these structures, defined by the BrU incorporation (Fig. 5E), as putative RV-B14 replication sites.

Triple labeling (BrU-RNA/Myosin Vb/RV-B14 capsid proteins) demonstrated the involvement of myosin Vb in the putative RV-B14 replication sites (Figs 6 and 7). According to the literature, overexpression of the myosin Vb tail domain led to the accumulation of early endosomal antigen 1 positive endosomes near myosin Vb clusters located in the vicinity of the nucleus^81^. Myosin Vb is required for transit out of plasma membrane recycling systems^32^, and the hijacking of its functions by RV-B14 infection could reroute the endosomal membranes to replication sites in an autophagosome-like manner^82^. If what we observed were replication sites (Figs. 5–7), the presence of myosin Vb could help to explain the diverse origins of the membranes found in replication sites of different enteroviruses, and the specific conjunct of proteins peculiar for each type of virus^83^.

Lastly, more tests are needed to identify the adaptor proteins that, together with MLCK, myosin Va, and Vb, support viral entry, genome release and interaction with the RNA clusters observed here that resemble viral replication sites.

## Materials and Methods

### Reagents

1 - (5 - Chloronaphthalenesulfonyl) homopiperazine - HCl (ML-9), Bromouridine (BrU), DMEM,, protease inhibitor cocktail, RNAse Inhibitor ProtectRNA™, and deoxynuclease I were obtained from Sigma (St. Louis, MO, USA). Fetal bovine serum (FBS), Opti-MEM, rabbit anti-BrdU, Alexa Fluor 555^®^ mouse anti-BrdU, rabbit anti-myosin Va, mouse anti-myosin Va, mouse anti-myosin Vb FITC, and Alexa Fluor 488^®^ goat anti-Mouse were from Life Technologies (Carlsbad, CA, USA). Pluronic F68 was from Merck (Rahway, NJ, USA). FuGENE HD was from Promega (Madison, WI, USA). IRDye^®^ 680LT Donkey anti-Rabbit IgG, IRDye^®^ 800LT Donkey anti-Rabbit IgG, and IRDye^®^ 680LT Donkey anti-Mouse IgG were from LI-COR Biosciences (Lincoln, NE, USA). Goat Anti-Rabbit IgG H&L (FITC), Goat Anti-Rabbit IgG H&L (Tetramethylrhodamine (TRITC)), and Goat Anti-Mouse IgG H&L (Agarose) were from ABCAM (Cambridge, UK).

### Cell Culture

Human cervix carcinoma cells expressing ICAM-1 (HeLa-H1, CRL 1958; ATCC, Manassas, VA) were maintained in suspension culture or as attached cells. For suspension cultures, cells were maintained in complete DMEM (8% FBS, 1 mM EDTA, 1% pluronic F68, and 1% penicillin/streptomycin) within Erlenmeyer flasks shaken at 100 rpm at 37 °C in a 5% CO_2_ atmosphere. Cultures were seeded at a concentration of 5 × 10^5^ cells/mL and subdivided every 3–4 days. Monolayer cell cultures were maintained in DMEM without phenol red supplemented with 10% FBS, 30 mM MgCl_2_, and 1% penicillin/streptomycin. Cells were subdivided before confluence.

### Virus production, purification, and metabolic labeling with BrU

Suspension cell cultures of HeLa-H1 were transferred to DMEM without phenol red supplemented with 1% FBS, 30 mM MgCl_2_, and 1% penicillin/streptomycin at 34 °C. RV-B14 was added at an multiplicity of infection (MOI) of 0.1 and cells remained in culture for 10 h. Cells (2 L) were separated from the media by centrifugation (1,000 × *g* for 5 min at 25 °C), resuspended in 5–10 mL DMEM without phenol red, transferred to 50 mL round bottom tubes, and subjected to five freeze-thaw cycles using liquid nitrogen and 20 °C water bath. Cell debris was removed by centrifugation (10,000 × *g* for 30 min) and the supernatants were combined. Sarkosyl (to 1%) and 10 mM EDTA were added prior centrifugation (200,000 × *g* for 2 h @ 4 °C). The pellet, containing virus, was suspended in TE buffer (10 mM Tris-HCl and 1 mM EDTA; pH 7.5), layered on top of a 5–45% discontinuous sucrose gradient made with TE buffer, and centrifuged (250,000 × *g* for 90 min @ 4 °C). Banded virus was detected in collected fractions by western blot analysis using anti-RV-B14 sera (see details below). Positive fractions were pooled and diluted with two volumes of TE buffer for storage at −70 °C. All virus fractions were tested for purity (electron microscopy and dynamic light scattering assay) and infectivity. Metabolic labeling of the viral genome with BrU (BrU-RV-B14) was accomplished by adding 10 mM BrU to the growth media at 30 min PI. Beside BrU pulse, the production of BrU-RV-B14 was the same for RV-B14 described above.

### Analysis of RV-B14 infectivity

The infectivity of RV-B14 virus samples was measured according to the 50% tissue culture infective dose (TCID_50_) using HeLa-H1 cells. Adherent cells were infected with serial dilutions of virus stocks ranging from 10^−1^ to 10^−8^, with the infection medium described previously. After 48 h incubation at 34 °C, the viability of the cells was measured via crystal violet staining. The TCID_50_ was calculated according to the method of Reed and Muench^84^.

### Experimental Infections of adherent HeLa-H1

Synchronized infections with RV-B14 were achieved by a 15 min pre-incubation of cells at 4 °C, addition of virus at designated MOIs, and an additional 15 min incubation at 4 °C. Next, cells were washed with ice-cold PBS, to remove nonattached virus particles, and internalization was initiated by the addition of pre-warmed DMEM without phenol red (35 °C). Infections were allowed to proceed as described in the text, and halted by transfer into a 3.8% formaldehyde solution.

### Plasmid expression vectors and cell transfections

The carboxy-terminal coding sequence (aa 955 - aa 1852) from the brain isoform of mouse myosin Va^85^ was amplified for fusion to the carboxy-terminus of eGFP in the mammalian expression vector pEGFP-C2 (Takara Bio, Mountain View, CA). The myosin Vb tail fused to eGFP was a gift from Jim Goldenring^32^. The GFP expression control used the mammalian expression vector pEGFP-C2 alone (Takara Bio, Mountain View, CA). Cells were grown on 20 mm glass coverslips until ~80% confluence and transfected (1 µg of DNA and 2 µL of FuGENE HD diluted in Opti-MEM). After 1 hour, complete growth medium was added to the plates. Experiments were performed 18 hours after transfection.

### Anti-RV-B14 antibody generation and immunolabeling

A high titer polyclonal sera was generated against RV-B14 according to the method described previously^86^ in partnership with the APCBiotecnologia (IBfCCF – UFRJ). Briefly, two rabbits were injected three times over the course of 45 days with 1 µg of high purity RV-B14. Two weeks after the last injection, total serum was obtained and filtered (0.22 µm, low-binding). Antibody titer was determined against RV-B14 by dot blot assay and antigenicity specificity was tested by western blot.

For immunofluorescence assays, cells were fixed with 3.8% formaldehyde in PBS, washed three times with PBS for 5 min each, permeabilized in PBS with 0.1% Triton X-100, washed three times in PBS, and blocked (5% BSA and 0.05% Tween 20 in PBS) for 1 h before incubation with antibodies. To detect myosin Va, myosin Vb, RV-B14 proteins, and BrU (according to each assay), a sequential labeling protocol was used. Initially, mouse anti-myosin Va or mouse anti-myosin Vb (1:500 dilution) were co-incubated with anti-RV-B14 rabbit sera (1:1000 dilution) for 1 h in PBS with 5% BSA and 0.05% Tween 20. Next, the coverslips were washed three times in PBS for 5 min followed by incubation with the appropriate fluorescent secondary antibody. After 1 h, the coverslips were washed three times with PBS and then incubated with the directly labeled antibody against BrU (1:250 dilution). Cells were prepared according Rietdorf^40^ to label virus outside the cell (Fig. 1A–C) and inside the cell (Fig. 1D–F). Finally, the coverslips were washed with PBS three times and mounted for microscopy.

### Confocal fluorescence microscopy and image analysis

Images of the fluorescent signals were acquired at room temperature with a LSM510 META Microscope (Carl Zeiss, Oberkochen, Germany) equipped with a 100X oil immersion objective (N.A. 1.42). Images were captured with the iris set for one airy unit and 1024 × 1024 pixel resolution. Following acquisition, images were quantified for various parameters as described in the text. In all instances, the pixel intensities were obtained from the raw images.

### Myosin Va immunoprecipitation and DOT blot assay

Cells were incubated in DMEM without phenol red supplemented with 1% FBS, 30 mM MgCl_2_, and 1% penicillin/streptomycin at 4 °C with BrU-RV-B14 (MOI = 10) for 15 min, to allow viral attachment. To start the infection, the medium was exchanged for pre-warmed medium (35 °C). Infections were continued for 0 (infection was stopped after virus attachment), 15, 30, 60, 90, 180 or 270 min. Samples were cross-linked with 1% formaldehyde for 2 min to halt the infection and to stabilize intracellular complexes. RIPA buffer was then added to the cells and protein-RNA complex extraction proceeded according to the RIP protocol^87^. Briefly, cells were treated with 200 mM glycine-PBS then washed with ice-cold PBS. Cells were kept on ice and scrapped into lysis buffer (50 mM HEPES-KOH pH 7.5, 140 mM NaCl, 1 mM EDTA pH 8, 1% Triton X-100, 0.1% sodium deoxycholate, 0.1% SDS, protease Inhibitor cocktail, RNAse Inhibitor ProtectRNA™, DNase I, and 1% BSA). Homogenate were collected and centrifuged (5,000 × *g* for 30 min at 4 °C). The supernatant was then incubated for 4 h with mouse anti-myosin Va antibody at 4 °C with rocking. Agarose beads coupled with anti-Mouse antibody were added for an overnight incubation with rocking at 4 °C. The next day, beads were collected by centrifugation and washed six times with lysis buffer. Precipitated complexes were released with elution buffer (6 M urea, 20 mM Tris-HCl pH 7.5, 100 mM NaCl) and samples (10 µg total protein) were subsequently applied by vacuum to a low-fluorescence PVDF membrane (LI-COR Biosciences, Lincoln, NE) with the aid of a Dot Blot Microfiltration Apparatus (BioRad, Hercules, CA, US). Membranes were washed and blocked with Odyssey Blocking Solution (LI-COR Biosciences) for 4 h. Next, the membrane was incubated with a solution containing rabbit anti-BrU antibody (1:1000 dilution) diluted in Blocking Buffer for 2 h, washed 3 times, and incubated with anti-Rabbit IRDye 800 nm antibody (1:5000 dilution) for 2 h. Membranes were washed and scanned with Odyssey (LI-COR Biosciences). Membranes were stripped using 10X EK-Away Stripping Buffer (Life Technologies, Carlsbad, CA), according to the manufacturer’s protocol, followed by incubation with loading control (mouse anti-myosin Va and anti-Mouse IRDye 680), washing, and scanning. Results are shown as bars of the anti-BrU (anti-Rabbit IRDye 800) signal normalized to the anti-myosin Va (anti-Mouse IRDye 680) signal.

### Ethics statement

Antibody generation by partnership with APC Biotecnologia (Instituto de Biofísica Carlos Chagas Filho - UFRJ) involving animals were carried out in accordance with the recommendations of the National Council for the Control of Animal Experimentation (CONCEA), and were approved by the institutional Ethics Committee on Animal Use (CEUA).

### Statistical analysis

Analyzes were performed using GraphPad Prisma 5.01 statistical software. The variables of the analyzed groups were compared using a non-parametric Kruskal Wallis ANOVA test. Multiple comparisons were made using Dunn’s multiple test. A probability value of less than 0.05 was regarded as significant.

### Data availability

All data generated or analyzed during this study are included in this published article (and its Supplementary Information files).

## Electronic supplementary material

Supplementary figure S1
