# Supplementary material for: Impairing the function of MLCK, myosin Va or myosin Vb disrupts Rhinovirus B14 replication
Source: Sci Rep. 2017 Dec 7;7:17153. doi: 10.1038/s41598-017-17501-z (PMC5719429; doi:10.1038/s41598-017-17501-z)
Supplement: Supplementary file 1 — Supplementary figure S1 [file 41598_2017_17501_MOESM1_ESM.pdf]

Impairing the function of MLCK, myosin Va or myosin Vb disrupts Rhinovirus B14 replication

Antonio Real Hohn[1,2,5], D. William Provance, Jr.[3], Rafael Braga Gonçalves[4,5], Caio Bidueira Denani[1,5], Andréa Cheble de Oliveira[1,5], Verônica P. Salerno[2] and Andre Marco de Oliveira Gomes[1,5*]

[1]Instituto de Bioquímica Médica Leopoldo de Meis, Universidade Federal do Rio de Janeiro, Rio de Janeiro, Brazil.

[2]Departamento de Biociências da Atividade Física, Escola de Educação Física e Desportos, Universidade Federal Rio do Janeiro, Brazil.

[3]Center for Technological Development in Health, National Institute of Science and Technology for Innovation in Diseases of Neglected Populations, Oswaldo Cruz Foundation/Fiocruz, Rio de Janeiro, Brazil.

[4]Departamento de Bioquímica, Instituto Biomédico, Universidade Federal do Estado do Rio de Janeiro, Rio de Janeiro, Brazil.

[5]Instituto Nacional de Ciência e Tecnologia de Biologia Estrutural e Bioimagem, Rio de Janeiro, RJ, Brazil.

* Corresponding author

E-mail: amog@bioqmed.ufrj.br.

Supplementary Figure S1:

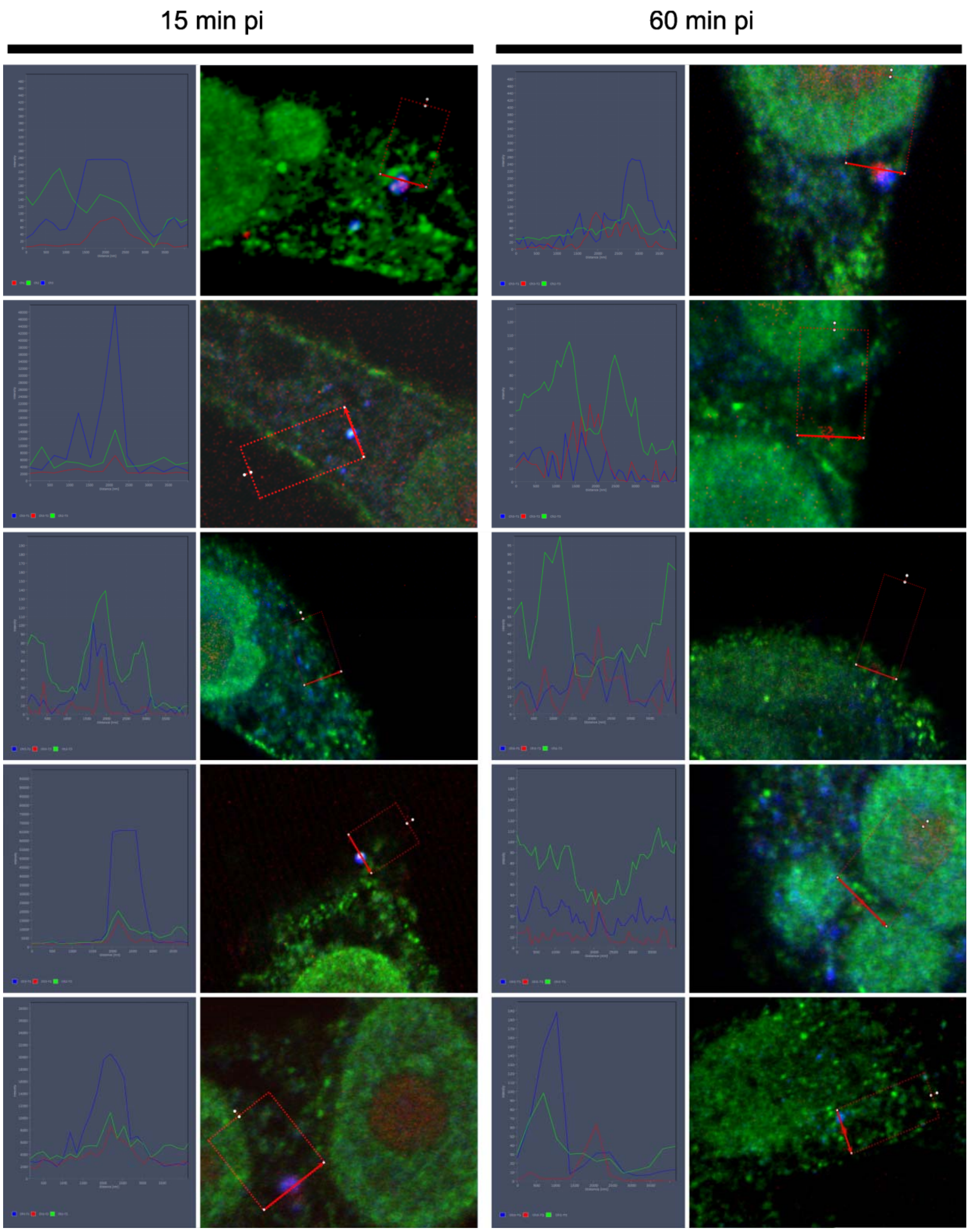

Supplementary Figure S1 Examples of intensitiy histograms used for Fig. 3Q and 3R. Images extracted from Zen Software (Carl Zeiss, Oberkochen, Germany). Experiments were conducted similarly as Fig. 3. Left column - cells at 15 min PI. Right column – cells at 60 min PI.
